# Adipose tissue-derived stem cell therapy in rat cryopreserved ovarian grafts

**DOI:** 10.1186/s13287-015-0068-3

**Published:** 2015-04-15

**Authors:** Luciana Lamarão Damous, Juliana Sanajotti Nakamuta, Ana Elisa Teófilo Saturi de Carvalho, José Maria Soares-Jr, Manuel de Jesus Simões, José Eduardo Krieger, Edmund C Baracat

**Affiliations:** Gynecology Discipline, Laboratory of Structural and Molecular Gynecology (LIM-58), Hospital das Clínicas da Faculdade de Medicina da Universidade de São Paulo, Dr Arnaldo av 455, 2nd floor, room 2113, Pacaembu, São Paulo 01246-903 Brazil; Laboratory of Genetics and Molecular Cardiology, Heart Institute (Incor), Faculdade de Medicina da Universidade de São Paulo, Dr Enéas de Carvalho Aguiar Av 44, 10th floor, Cerqueira Cesar, São Paulo 05403-000 Brazil; Department of Morphology and Genetics, Universidade Federal de São Paulo (UNIFESP), Botucatu St 740. Ed. Lemos Torres, 2nd floor, Vila Clementino, São Paulo 04023-009 Brazil; Galvão Bueno St, 499, Bloco A. Apto31, Liberdade, São Paulo 01506-000 Brazil

## Abstract

The preliminary results of ovarian transplantation in clinical practice are encouraging. However, the follicular depletion caused by ischemic injury is a main concern and is directly related to short-term graft survival. Cell therapy with adipose tissue-derived stem cells (ASCs) could be an alternative to induce early angiogenesis in the graft. This study aimed to evaluate ASCs therapy in rat cryopreserved ovarian grafts. A single dose of rat ASC (rASCs) or vehicle was injected into the bilateral cryopreserved ovaries of twelve adult female rats immediately after an autologous transplant. Daily vaginal smears were performed for estrous cycle evaluation until euthanasia on postoperative day 30. Follicle viability, graft morphology and apoptosis were assessed. No differences were found with respect to estrous cycle resumption and follicle viability (P > 0.05). However, compared with the vehicle-treated grafts, the morphology of the ASCs-treated grafts was impaired, with diffuse atrophy and increased apoptosis (P < 0.05). ASCs direct injected in the stroma of rat cryopreserved ovarian grafts impaired its morphology although may not interfere with the functional resumption on short-term. Further investigations are necessary to evaluated whether it could compromise their viability in the long-term.

**Electronic supplementary material:**

The online version of this article (doi:10.1186/s13287-015-0068-3) contains supplementary material, which is available to authorized users.

## Findings

### Background

Despite the reestablishment of the endocrine function after ovary transplantation, follicular depletion caused by an ischemic lesion is a main concern and is directly related to short-term graft survival [1-5]. The challenges to be met in the next few years should include a focus on the improvement of cryopreservation techniques and on host bed conditions [3]. Cell therapy with adipose tissue-derived stem cells (ASCs) may be a viable and advantageous alternative to current approaches.

The therapeutic potential of ASCs has been demonstrated in a number of preclinical models. When there is a lesion, ASCs lessen tissue damage, inhibit fibrotic remodeling and apoptosis, promote angiogenesis, stimulate endogenous stem cell recruitment and proliferation, and reduce the immune response [6]. Studies with animal models are necessary to understand the induction of angiogenesis in an ischemic environment and to ensure that treatment with such cells is safe [7].

Improvements in rat ovarian function have been achieved by the direct injection of ASCs into ovaries damaged by chemotherapy drugs [8,9]. However, few data are currently available on the therapeutic potential of ASCs in ovarian transplantation. An initial assessment was carried out to evaluate the feasibility and safety of this line of inquiry, in which rat ASCs transgenic for the green fluorescent protein (GFP) were injected into fresh rat grafts. ASCs were found to remain viable in ovarian tissue without inducing morphological changes or functional damage; some even assumed an endothelial phenotype and promoted early resumption of the estrous cycle and an increase in angiogenesis (LLD, unpublished observations). Nevertheless, there is not enough current knowledge of ASCs actions in cryopreserved grafts. For this reason, this study aimed to determine whether ASCs therapy could improve the viability of cryopreserved ovarian grafts, allowing for translational results.

## Methods

The study was carried out at the Laboratory of Structural and Molecular Gynecology (LIM-58), Gynecology Discipline, Department of Obstetrics and Gynecology, Hospital das Clínicas da Faculdade de Medicina da Universidade de Sao Paulo (FMUSP), in cooperation with the Laboratory of Genetics and Molecular Cardiology/Heart Institute/FMUSP. The experimental procedures followed the institutional guidelines for the care and use of laboratory animals and were approved by the Institutional Ethics Committee/FMUSP, protocol 190/10, approved on 14 April 2011. This study was initiated in July 2013 and terminated in January 2014.

The study sample consisted of 12 twelve-week-old adult female Wistar (*Rattus norvegicus albinus)* rats. The animals had access to a breed-specific food formula and water *ad libitum* throughout the experiment and were kept under adequate sanitary, lighting (12 hour), and temperature conditions in the animal laboratory. Before the experiment, vaginal smears were obtained daily. Only those animals showing at least two consecutive normal four-day vaginal estrous cycles were included in the experiment. The ovarian transplant was performed during the diestrous phase.

Rat ASCs (rASCs) obtained from the inguinal region of Wistar rats or vehicle were injected into bilateral ovarian grafts of 12 Wistar rats. An ovarian graft is the whole frozen-thawed ovary grafted into the retroperitoneum, one on each side of the psoas muscle, without vascular anastomosis.

### ASCs isolation and ex vivo expansion

Inguinal subcutaneous adipose tissue was collected under sterile conditions from ten-week-old male Wistar rats and was rinsed with phosphate-buffered saline (PBS). ASCs were isolated, characterized, and maintained in culture as previously described [10]. Details are available in Additional file 1 (ASCs isolation and ex vivo expansion).

The morphological and replicative characteristics and the immunophenotypes (CD90^+^, CD29^+^, CD44^+^, CD73^+^, CD31^−^, CD45^−^) of the ASCs have been previously determined in our laboratory [11]. Despite the fact that the characterization had been done extensively in mice adipose-derived stem cells (mASC), our group conducted some immunophenotyping assays with rASCs. Accordingly, the percentage of CD90 and CD29 positive cells, the main markers of a variety of adult stem cells, was found to be around 90% (92, 65% and 98, 89%, respectively) in rASC at the third culture passage (JSN *et al*., unpublished observations).

### Operative procedures

The rats were anesthetized intraperitoneally with xylazine and ketamine. After bilateral oophorectomy, the fresh ovaries were immediately frozen in a slow-cooling freezer (CL-8800, Cryogenesis software, Freezer Control) as previously described [12]. Details are available in Additional file 2 (ovarian cryopreservation). A second laparotomy was performed 24 hours after ovarian cryopreservation. Each animal received a pair of autologous ovary transplants. With a simple stitch of 4–0 nylon suture, intact whole ovaries were implanted in the retroperitoneum, one on each side of the psoas muscle, in proximity to the aorta and vena cava, without vascular anastomosis.

At this point, the rats were distributed into two experimental groups of six animals each, according to treatment, as follows: vehicle (low glucose DMEM) or ASCs at a concentration of 5 × 10^4^ cells in 25 μl of vehicle/ovary. The technical standardization of ASCs injection was performed according to a pilot project conducted in the intact ovary (topic) in which ASCs obtained from rats transgenic for GFP (ASC-GFP^+^) were injected. In this, we tested different vehicle volumes for cell delivery (ranging from 2 to 50 μl) with the aid of a surgical microscope. The standard dose of 25 μl was the lowest in which no leakage was visually observed following the injection, without changes in ovary morphology and ASC-GFP^+^ were easily identified and quantified in ovarian stroma (Data not shown).

The treatment was injected into both ovarian grafts with only one shot into the center of the ovarian parenchyma. No leakage was visually observed after injection. The procedures were carried out with the aid of a surgical microscope (16x).

Beginning on postoperative (PO) day 4, vaginal smears were obtained from each rat between 8:00 a.m. and 10:00 a.m. daily until euthanasia, which was performed between day 30 and day 35, always when the rats were in diestrous phase. The animals underwent a third surgical procedure and the grafts were subsequently removed whole.

One ovarian graft was immediately fixed in 4% paraformaldehyde for at least 24 hours. After fixation, the ovaries were dehydrated, paraffin-embedded, serially sectioned at 5 μm, and mounted on glass microscope slides. Routine hematoxylin and eosin (H & E) staining was performed for histologic examination via light microscopy. The other ovarian graft was prepared for the follicular viability assay. After this procedure, the animals were euthanized with a lethal dose of the previously used anesthetics.

### Morphological analyses

Morphological evaluation was achieved through descriptive analyses of the grafts. Assessment of follicular quality was based on cell density, the presence or absence of pyknotic bodies, and the integrity of the basement membrane and of the oocyte. According to these criteria, follicles were classified as normal or degenerated; only the former were characterized and quantified. Viable follicles were classified as previously described [13].

### Follicular viability assay

Using a modified protocol [14], the analysis of the follicle viability was carried out as detailed in Additional file 3 (follicular viability assay). A three-fold manual count of each well was performed under an inverted microscope (EVOS® XL Core Cell Imaging System*,* AMG) at 200× magnification to allow for individualization of the white (viable) and blue (atretic) follicles in absolute numbers as well as in percentages of viable follicles.

### Apoptosis assay

Sections containing ovarian stroma were immunostained to measure apoptosis via cleaved-caspase-3 expression (SANT-SC-1226, 1:100, Santa Cruz Biotechnology, Inc. Santa Cruz, CA, EUA) and the terminal deoxynucleotidyl transferase (TdT)–mediated dUTP nickend labeling (TUNEL) assay using a commercially available kit (In Situ Cell Death Detection Kit, Fluorescein, Roche, Berlin, Germany, 11684795910) following the manufacturer’s instructions. Red-brown coloring of the cytoplasm/nucleus of the granulosa cells was considered positive staining (any other coloring was considered negative staining). For the negative controls, the primary antibody was omitted to avoid bias.

Images of the sections were obtained using an image acquisition software system (Leica DM2500); measurements were made using the Leica QWin V3 software. Red-brown coloring of the cytoplasm/nucleus of the stroma cells was specified as positive staining (anything else as negative staining). A positive cell staining assessment was performed in four different fields per animal at 200x magnification and the results are expressed as a percentage of the positive area (arbitrary unity/mm^2^). Two independent investigators blinded to the experimental treatments performed all measurements.

### Statistical analysis

The results were expressed as the mean ± standard deviation of the mean. The unpaired t-test was utilized to compare groups (vehicle and ASCs). All statistical analyses were performed using Graphpad Prism 4.0 (Graphpad Software Inc, CA, San Diego, USA). *P* values less than 0.05 were considered significant.

## Results

### Estrous cycle resumption after transplantation

All animals resumed cycling after transplantation, and the PO day of estrous phase identification was similar for all treatment groups (vehicle: 16.66 ± 2 versus ASCs: 18.66 ± 5.77, *P* >0.05).

### Morphology and follicular viability of the grafts 30 days after transplantation

**Figure 1:**
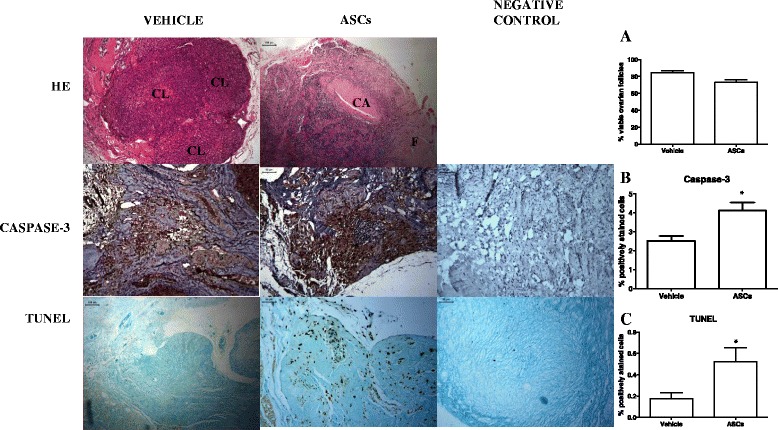
Photomicrography of cryopreserved ovarian grafts treated with vehicle or ASC 30 days after an autologous avascular transplant. The morphology of the ASCs-treated grafts shows increased fibrosis and more corpora albicans, but the follicle viability was maintained, as evidenced by trypan blue **(A)** (*P* >0.05). Immunohistochemistry for apoptosis was performed on the ovarian stroma, and dark brown-stained cells are considered positive. Results are expressed as a percentage of the positive area (arbitrary unity/mm^2^). Apoptosis increased in both analyses (cleaved-caspase-3 and TUNEL) of the ASC-treated grafts **(B and C)** (*P* <0.05). H & E 100x. Immunohistochemistry analysis 200x. ASCs, adipose tissue-derived stem cells; CL, corpora lutea; CA, corpora albicans; F, fibrosis. * *P* <0.05, unpaired t-test.

In the vehicle-treated animals, ovarian follicles were observed at various stages of maturation, from primordial follicles to preovulatory follicles, occasionally with luteinization on the wall. Corpora lutea and occasional corpora albicans were detected. In the ASCs-treated animals, the ovarian grafts displayed diffuse atrophy and corpora albicans. The vascular network exhibited thickening and hyalinization of the blood vessel walls associated with the obliteration of the lumen. The presence of gigantocellular reactions of the foreign body type to the suture was also observed. The percentage of viable ovarian follicles by trypan blue was similar between groups (vehicle: 85.2 ± 6.8 versus ASCs: 74.5 ± 10.3, *P* >0.05) (Figure 1, Table 1).

**Table 1:** **Ovarian follicles after isolation and trypan blue staining, according to treatment**

**Rats**	**Vehicle**			**ASCs**		
	**Number unstained**	**Number blue**	**Viable follicles (%)**	**Number unstained**	**Number blue**	**Viable follicles (%)**
R1	60	12	83.33	45	12	78.94
R2	83	20	80.58	73	11	86.9
R3	67	8	89.33	35	7	83.33
R4	46	3	93.87	42	28	60
R5	49	6	89.09	52	30	72.22
R6	54	18	75	33	50	66
Mean	59.8 ± 12.4	11.1 ± 6.1	85.2 ± 6.2	46.6 ± 13.3	23 ± 14.8	74.5 ± 9.4

Data are shown as mean number (±Standard Deviation), *P*>0.05, paired t-test.

### Apoptosis assay

The ASCs treatment boosted apoptosis as measured both by the cleaved-caspase-3 (vehicle: 2.52 ± 0.73 versus ASCs: 4.11 ± 1.07, *P* <0.05) and the TUNEL (vehicle: 0.17 ± 0.18 versus ASCs: 0.52 ± 0.46, *P* <0.05) methods (Figure 1).

## Discussion

The ASCs-treated cryopreserved ovarian grafts maintained the percentage of viable ovarian follicles and did not alter the functional resumption of the graft, as evidenced by the estrous phase resumption in the vaginal smears. The morphology of the ASCs-treated grafts, however, was more impaired, as indicated by more fibrosis, a predominance of corpora albicans, and increased apoptosis. In the long-term, this therapy might negatively affect the viability of cryopreserved ovarian tissue. The direct injection of ASCs may have overstimulated the intrinsic inflammatory response in an environment already under adverse hypoxic conditions. Our experimental model has some limitations: 1) clinical application is not possible due to the rat ovary being different from the human one; and 2) further long-term study with fresh grafts is necessary to confirm our results.

Another relevant point is the type of lesion induced in the ovary. In ovaries damaged by chemotherapy drugs, the ASCs treatment promoted functional recovery and mitigated the lesions [8,9]. Further, fresh ovarian grafts showed early functional resumption with no changes in apoptosis (LLD, unpublished observations). In the present model, ovaries cryopreserved by slow freezing are biologically different from fresh tissue, which has only undergone oophorectomy, or from tissue severely impaired by chemotherapy drugs. Cryoinjury has been exhaustively investigated in human and animal tissues because of the attainable translational results. The current consensus is that ischemia prior to graft revascularization rather than cryopreservation-induced damage is the main cause of follicular loss [2,4]. It cannot be stated, however, that cryopreservation is completely inert concerning the ovarian tissue [13,15-18].

In clinical practice, long-term follow-up is still scarce. The slow-freezing protocol has been used in every case of a baby born from ovary transplantation to date [3]. A recent literature review showed that the pattern of functional resumption was the same for fresh and cryopreserved grafts. However, the survival of the cryopreserved grafts was two-thirds shorter than that of the fresh grafts [19], a fact that may be due to the intrinsic problems of slow freezing. Thus, the development of improved ovarian tissue freezing techniques for transplantation is a current priority to increase the success rates of the technique [3].

As ASCs were observed to remain viable in ovarian tissue for 30 days after a single injection into the center of the parenchyma (LLD, unpublished observations), we believe this therapy should be further investigated in a translational model with cryopreserved ovaries despite the increase in apoptosis found in this study. We assume that the direct injection of ASCs into the cryopreserved ovarian tissue with attendant exacerbation of the local inflammatory response may have been an overly aggressive treatment. Therefore, it is necessary to develop new strategies for delivering ASCs.

ASCs are usually administered by systemic injection (intravenous or intra-arterial) or local injection into damaged tissues. These administration methods may affect cell viability and the dose actually delivered. Counterpressure and tissue density should be taken into account to control for the cell dose and to maintain the structural integrity of the cells [20]. Another option is to deliver the ASCs through an acellular matrix. This strategy has several advantages over a direct injection, such as allowing prolonged contact with the target tissue, prompting cell retention, and furthering the gradual and continuous release of proangiogenic factors [21]. This being the case, the next technical challenges that must be overcome in ASCs treatment are how to reach the target tissue with precision and how to optimize ASCs action to keep the cells viable in large enough quantities and for a long enough time to trigger the expected therapeutic action.

## Conclusions

ASCs directly injected in the stroma of rat cryopreserved ovarian grafts impaired its morphology although they may not have interfered with the resumption of its function in the short-term. Further investigations are necessary to evaluate whether it could compromise their viability in the long-term.

## Additional files

Additional file 1:
**ASC isolation and** ***ex vivo*** **expansion.**
Additional file 2:
**Ovarian cryopreservation.**
Additional file 3:
**Follicular viability assay.**

## Abbreviations

ASCs: adipose tissue-derived stem cells
(D)MEM: (Dulbecco’s) modified Eagle’s medium
DMSO: dimethyl sufoxide
FBS: fetal sovine Serum
GFP: green fluorescent protein
H & E: hematoxylin and eosin
PBS: phosphate-buffered saline
PO: postoperative
rASCs: rat ASCs
TUNEL: deoxynucleotidyl transferase (TdT)–mediated dUTP nickend labeling
