# Supplementary material for: Adipose tissue-derived stem cell therapy in rat cryopreserved ovarian grafts
Source: Stem Cell Res Ther. 2015 Apr 15;6(1):57. doi: 10.1186/s13287-015-0068-3 (PMC4416311; doi:10.1186/s13287-015-0068-3)
Supplement: Additional file 1: — ASC isolation and ex vivo expansion. [file 13287_2015_68_MOESM1_ESM.pdf]

ASC isolation and *ex vivo* expansion

The harvested tissue was dissociated by digestion with 0.075% type IA collagenase (Sigma-Aldrich, Inc.) for 45 minutes. Enzyme activity was stopped and the cell suspension was centrifuged at 300g for 15minutes. Pelleted cells were recovered and plated onto 10-cm culture plates (NUNC, Rochester, NY). At 24-hour intervals, cultures were washed with PBS to remove contaminating erythrocytes and other unattached cells, and then reefed with fresh medium. The plating and expansion medium consisted of low glucose Dulbecco's modified Eagle's medium (DMEM) supplemented with 10% Fetal Bovine Serum (FBS) and penicillin/streptomycin antibiotics (Invitrogen Corporation, Carlsbad, CA).

Cells were maintained at 37ºC with 5% CO_2 in tissue culture dishes and fed twice a week until they reached 80% of confluence - usually within 5 to 7 days after the initial plating. Once 80% confluence was reached (passage 0), adherent cells were detached with 0.25% trypsin-EDTA (Vitrocel Embriolife, Campinas, SP, Brazil) and were either replated at $1x10^4$cells/cm^2 or used for experiments. Cultures were passaged every 3 to 5 days and used for experimental procedures until passage 3.
