# Supplementary material for: Adipose tissue-derived stem cell therapy in rat cryopreserved ovarian grafts
Source: Stem Cell Res Ther. 2015 Apr 15;6(1):57. doi: 10.1186/s13287-015-0068-3 (PMC4416311; doi:10.1186/s13287-015-0068-3)
Supplement: Additional file 2: — Ovarian cryopreservation. [file 13287_2015_68_MOESM2_ESM.pdf]

## Ovarian cryopreservation

The whole ovaries were placed in 1.2ml-cryovials (Sigma-Aldrich®, Inc.) in M2 medium plus HEPES without Penicilin and Streptomycin (M2-Sigma-Aldrich®, Inc.) and with 1.4M dimethyl sulfoxide (DMSO) (Sigma-Aldrich®, Inc.) as a cryoprotector and were held at room temperature for 5 min. The cryovials were sealed by twisting their caps and were placed in a temperature-programmed freezer (CL-8800, Cryogenesis software, Freezer Control) and cooled from 25$^{o}$C to 10$^{o}$C at a rate of 1$^{o}$C/min, then to -7$^{o}$C at a rate of 0.5$^{o}$C/min, and maintained at -7$^{o}$C for 5 min. Then, ice nucleation was manually induced using pre-cooled forceps, and the temperature was held at -7$^{o}$C for another 5 min to allow for the release of latent heat fusion. The tissue was then frozen at -55$^{o}$C at a rate of 0,5$^{o}$C/min, plunged into liquid nitrogen at -196$^{o}$C and stored for 24 hours.

For thawing, cryovials were removed from liquid nitrogen and held at room temperature until the ice melted. The ovaries were washed two times for 5 min in fresh M2 medium, then shaken gently to remove the cryoprotectant before further processing. The ovaries were maintained in M2 at room temperature until the transplant.
