# Supplementary material for: Adipose tissue-derived stem cell therapy in rat cryopreserved ovarian grafts
Source: Stem Cell Res Ther. 2015 Apr 15;6(1):57. doi: 10.1186/s13287-015-0068-3 (PMC4416311; doi:10.1186/s13287-015-0068-3)
Supplement: Additional file 3: — Follicular viability assay. [file 13287_2015_68_MOESM3_ESM.pdf]

Follicular viability assay

The ovaries were washed with M2, sectioned with a scalpel, and transferred to a 50-ml tube containing 1 mg/ml (200 IU/ml) of type Ia collagenase solution (Invitrogen Corporation) and 2 ml of Leibovitz L-15 medium supplemented with L-glutamine and penicillin/streptomycin antibiotics (Invitrogen Corporation, Carlsbad, CA) to further dissolve the tissue further and make the follicles easy to count. This material was incubated in an oven at 37°C for 1 hour and slightly agitated every 10 minutes. The tissue digestion was interrupted with the addition of 1 ml of fetal bovine serum (Gibco). The solution was then filtered using a 100-µm nylon strainer (Cell Strainer 100-µm nylon, BD Falcon, Belgium) and centrifuged at 1500 rpm for 5 minutes at 20°C. The pellet was resuspended in 1ml of fetal bovine serum and the resulting suspension was placed in a 12-well plate (one ovary/well) to be stained with 20 µl of 0.4% trypan blue (Life Technologies) at room temperature for 10 minutes.
